# Exploring AI-2-mediated interspecies communications within rumen microbial communities

**DOI:** 10.1186/s40168-022-01367-z

**Published:** 2022-10-07

**Authors:** Xiaozhen Liu, Qinmeng Liu, Sihuai Sun, Hengxi Sun, Yao Wang, Xihui Shen, Lei Zhang

**Affiliations:** 1 Department of Microbiology and Bioengineering, College of Life Sciences, Northwest A&F University, Yangling, 712100 Shaanxi China; 2 State Key Laboratory of Crop Stress Biology for Arid Areas, Shaanxi Key Laboratory of Agricultural and Environmental Microbiology, College of Life Sciences, Northwest A&F University, Yangling, 712100 Shaanxi China; 3 Department of Biotechnology, College of Life Sciences, Northwest A&F University, Yangling, 712100 Shaanxi China

**Keywords:** Rumen, Microbes, Quorum sensing, AI-2, The CahR-type AI-2 receptors

## Abstract

**Background:**

The rumen is an ecosystem with a complex microbial microflora in which microbes initiate biofilm formation by attaching to plant surfaces for plant degradation and are capable of converting feed to nutrients and energy via microbial processes. Quorum sensing (QS) is a cell-to-cell communication mechanism that allows microbes to synchronize the expression of multiple genes in the group to perform social behaviors such as chemotaxis and biofilm formation using self-synthesized QS signaling molecules. Whereas QS has been extensively studied in model microorganisms under pure culture conditions, QS mechanisms are poorly understood in complex bacterial communities, such as the rumen microflora, in which cell-to-cell communication may be common.

**Results:**

Here, we analyzed 981 rumens bacterial and archaeal genomes from the Joint Genome Institute (JGI) and GenBank databases and identified 15 types of known QS signaling molecule-related genes. The analysis of the prevalence and abundance of genes involved in QS showed that 767 microbial genomes appeared to possess QS-related genes, including 680 bacterial genomes containing autoinducer-2 (AI-2) synthase- or receptor-encoding genes. *Prevotella*, *Butyivibrio*, *Ruminococcus*, *Oribacterium*, *Selenomonas*, and *Treponema*, known abundant bacterial genera in the rumen, possessed the greatest numbers of AI-2-related genes; these genes were highly expressed within the metatranscriptome dataset, suggesting that intra- and interspecies communication mediated by AI-2 among rumen microbes was universal in the rumen. The QS processes mediated by the dCache_1-containing AI-2 receptors (CahRs) with various functional modules may be essential for degrading plants, digesting food, and providing energy and nutrients to the host. Additionally, a universal natural network based on QS revealed how rumen microbes coordinate social behaviors via the AI-2-mediated QS system, most of which may potentially function via AI-2 binding to the extracellular sensor dCache_1 domain to activate corresponding receptors involved in different signal transduction pathways, such as methyl-accepting chemotaxis proteins, histidine kinases, serine phosphatases, c-di-GMP synthases and phosphodiesterases, and serine/threonine kinases in the rumen.

**Conclusions:**

The exploration of AI-2-related genes, especially CahR-type AI-2 receptors, greatly increased our insight into AI-2 as a potentially “universal” signal mediating social behaviors and will help us better understand microbial communication networks and the function of QS in plant-microbe interactions in complex microecosystems.

Video Abstract

**Supplementary Information:**

The online version contains supplementary material available at 10.1186/s40168-022-01367-z.

## Background

The rumen represents a bioreactor of the natural environment and converts the energy stored in plants that are indigestible by humans into digestible food products [1]. It contains a highly specialized, complex microbial community with a high-cell density (10^7^–10^8^ cells per kilogram) composed of bacteria, archaea, fungi, and viruses. These microbes usually attach to solid feed particles and form biofilms to achieve the degradation of various kinds of feed particles, thereby providing the host with energy and nutrients [2, 3]. However, progress in understanding rumen microbial communications has been slow due to the complexity of the ecosystem in the rumen. In-depth explorations of rumen microbial communication must be meaningful to increase the efficacy of plant degradation and ruminant production in the rumen microecological environment.

Quorum sensing (QS) is a cell-to-cell communication process mediated by autoinducers (AIs) that enables bacterial populations to coordinate their behaviors in a cell density-dependent manner [4, 5]. AIs are specific QS signaling molecules synthesized by microbial species and are widespread in prokaryotic cells [6]. When microbial community size becomes sufficiently large and the concentration of AIs reaches the threshold level required for detection, the AIs are bound by the corresponding receptors to trigger a signal transduction cascade for regulating the expression of target genes, causing a variety of bacterial behaviors, such as motility, biofilm formation, virulence factor expression, extracellular protease production, and antibiotic resistance [7, 8]. The known AIs that regulate physiological functions based on QS include *N*-acyl homoserine lactones (AHLs), autoinducing peptides (AIPs), autoinducer-2 (AI-2), diffusible signal factors (DSFs)/*Burkholderia cenocepacia* diffusible signal factors (BDSFs), indoles [9], autoinducter-3 (AI-3), cholerae autoinducer-1 (CAI-1) [10], diketopiperazines (DKPs) [11], *Pseudomonas* quinolone signal (PQS)/2-heptyl-4-quinolone (HHQ), 2-(2-hydroxyphenyl)-thiazole-4-carbaldehyde (IQS) [12], pyrones (PPYs) [13], dialkylresorcinols (DARs)/cyclohexanediones (CHDs), competence stimulating peptide (CSP) [14], 3,5-dimethylpyrazin-2-ol (DPO) [15], and 3-hydroxypalmitic acid methyl ester (3-OH-PAME)/Methyl 3-hydroxymyristate (3-OH-MAME). Three of these AIs have been described as major signaling molecules in the rumen thus far. In 2002, AHLs were detected in rumen fluid [16], providing evidence that AHLs may function in cell-to-cell communication in the rumen ecosystem. Subsequently, histidine kinase sensors identified as AIP-related protein components were also detected in bovine rumen contents [17]. In addition, AI-2 activity was observed in rumen contents and monospecies cultures of rumen bacteria such as *Butyrivibrio fibrisolvens*, *Eubacterium ruminantium*, *Ruminococcus flavefaciens*, and *Succinimonas amylolytica* [18, 19]. The AI-2 synthetase-encoding gene *luxS* was annotated in transcriptome data [20] and genome data [21] from rumen microbes, suggesting important roles of AI-2-based QS in the rumen. Recently, it has become increasingly difficult to ignore the functions of QS in complex microbial communities. However, the mechanisms of QS and functional QS genes involved in the rumen microbiota are still largely elusive. There remains a gap in the knowledge of how microbes use QS signaling molecules to communicate with each other and modify the social activities of the rumen microbiota, despite the advances in “omic” technologies, and this topic warrants further investigation.

The AI-2 family is a group of 4,5-dihydroxy-2,3-pentanedione (DPD) derivatives that are generally biosynthesized from S-ribosylhomocysteine (SRH) by the enzyme LuxS and serve as signaling molecules dedicated to intra- and interspecies communications among prokaryotic species [22]. To date, three types of AI-2 receptors have been found, including periplasmic binding proteins homologous to LuxP and LsrB and the recently reported dCache_1 domain-containing transmembrane AI-2 Receptor (hereafter referred to as CahR) [5, 22]. In general, LuxP is present only in *Vibrio* spp., and AI-2 binds to LuxP to trigger a signal transduction cascade [4], whereas LsrB directly binds AI-2 and delivers AI-2 into cells via the membrane members of the ATP-binding cassette transporter system Lsr to regulate density-dependent gene expression [23]. CahR-type AI-2 receptors potentially perform as extracytoplasmic sensors of transmembrane signal transduction proteins such as methyl-accepting chemotaxis proteins (MCPs), histidine kinases (HKs), c-di-GMP synthases and phosphodiesterases (GCDs), serine/threonine kinases (STKs), serine phosphatases (SPs), and adenylate- or guanylate cyclases (ACs/GCs) to induce the expression of downstream genes. For example, AI-2 binds to the dCache_1 of *P. aeruginosa* chemoreceptors PctA and TlpQ to induce biofilm formation and chemotaxis [22]. However, there are no studies related to AI-2 receptors based on QS in rumen microbes.

Here, we sought to analyze the lacunae in the knowledge of QS in rumen microbes to achieve a better understanding of different communication patterns that exist in nature. We systematically analyzed rumen microbial genomes and metatranscriptomes for genes/proteins involved in cell-to-cell communications, and 8 types of QS signaling molecules were observed in the rumen, suggesting the significance of the QS system in the rumen and indicating that AI-2 may mediate the diverse interspecies communications among rumen microbes. The diversity of AI-2 receptor functional modules and their high expression within the largest available rumen metatranscriptome datasets suggests that these sensors are widely distributed and function in rumen bacteria, thus expanding our understanding of the role of QS in modulating the functions of natural microbial communities. In addition, we determined the natural network of the microbes involved in cell-to-cell communication coordinating social behaviors in the rumen.

## Methods

### Microbial genomes

The rumen microbial genomes employed in this study were obtained from Shi et al. (501), Gharechahi et al. (538), and GenBank (https://www.ncbi.nlm.nih.gov/genome) (December 2020) (20) [24, 25]. The genomes in GenBank were searched with the keywords (“rumen and archaea” or “rumen and bacteria”), and duplicate genomes were removed. The remaining data were used for subsequent analysis. A total of 2809 metagenome-assembled genomes (MAGs) from the rumen ecosystem including cattle, sheep, moose, deer, and bison [26] and 6339 MAGs from the pig gut ecosystem [27] were obtained from publicly available datasets as the control group. Genome completeness and contamination were assessed using CheckM with the default settings [28].

### Rumen microbial QS-related proteins

The genomes were reannotated using PROKKA with the default settings, and the predicted proteins were searched by using semantic approaches and hmmsearch [29, 30]. To search for AHLs, AIPs, indoles, DSFs/BDSFs, AI-3, CAI-1, DKPs, PQS/HHQ, IQS, PPYs, DARs/CHDs, CSP, DPO, and 3-OH-PAME/3-OH-MAME-related proteins, the predicted related protein names were searched by semantic approaches (Table S1). To search for AI-2-related proteins, we performed a comparison of protein sequence and profile HMMs (S-Ribosylhomocysteinase LuxS (http://pfam.xfam.org/family/PF02664), the Autoinducer 2-binding protein LsrB/Autoinducer 2-binding periplasmic protein LuxP (http://pfam.xfam.org/family/PF13407), and the dCache_1 domain (http://pfam.xfam.org/family/PF02743)) with hmmsearch and searched the following related protein names: S-ribosylhomocysteine, S-ribosylhomocysteine lyase, LuxS, LsrB, and LuxP. All predicted dCache_1 domain sequences were aligned with reference sequences (PctA-LBD of *P. aeruginosa*) using hmmsearch. The dCache_1 domains with the five conserved amino acid residues R126, W128, Y144, D146, and D173 in PctA-LBD served as putative dCache_1 domain-containing AI-2 receptors [22, 31], and the predicted protein names were corrected based on the annotation of their protein domains (Pfam: Home page (xfam.org)) [32].

### AI-2 binding assays in vitro

To express and purify His_6_-tagged recombinant proteins, pET-28a derivatives carrying the dCache_1 domain DNA fragments were transformed into *Escherichia coli* BL21 (DE3) or its *luxS* gene deletion mutant strain. Bacteria were grown in LB medium at 37 °C for 10 h and reinoculated with a ratio of 1:100 into fresh LB at 37 °C to an OD_600_ of 0.8. Cultures were further grown at 20°C for 7 h with 0.25 mM IPTG at a speed setting of 150 rpm in a rotary shaker. Cells were collected, resuspended, and disrupted by sonification and then purified with the His-Bind Ni-NTA resin according to the manufacturer’s instructions (Novagen, Madison, WI). The purified proteins were eluted using a solution (300 mM NaCl, 50 mM NaH_2_PO_4_, and 250 mM imidazole) and then swapped into a solution (300 mM NaCl, 50 mM NaH_2_PO_4_, and 1 mM dithiothreitol). The proteins were further verified by SDS-PAGE analysis, concentrated to ~10 mg ml^−1^ and then denatured by heating for 10 min at 70 °C. For accessing AI-2 activity, *Vibrio harveyi* MM32 (LuxN^-^, LuxS^-^) of overnight culture was diluted 5000 times with AB fresh medium and 90 μL of MM32 diluted bacteria and10 μL of AI-2 supernatant from denatured proteins were mixed at 30 °C for 8 h in dark to measure the bioluminescence (counts per second) at a wavelength of 490 nm via microplate reader Victor X3 (PerkinElmer, Waltham, MA, USA). A buffer control was used as a negative control [22].

### Visualization of conserved amino acid residues

All putative dCache_1 domain sequences with five conserved amino acid residues in the rumen microbial genomes were aligned by ClustalW embedded in MEGA X software. Aligned columns which were absent in the dCache_1 domain of PctA were removed, and sequence logos were created using the WebLogo 3 online software (http://weblogo.threeplusone.com). The number of amino acids was based on the sequence of PctA-LBD of *P. aeruginosa.*

### The domain architecture

The domains of all putative CahR-type AI-2 receptor proteins were identified by multiple sequence alignment and profile hidden Markov models (HMMs) in a database of protein domain families (Pfam Database) with significant Pfam-A Matches (Pfam: Home page (xfam.org), May 2021).

### The construction of a phylogenetic tree

The putative dCache_1 domains from the CahR-type AI-2 receptors determined from the rumen bacteria were aligned with four references (*Pseudomonas aeruginosa* PAO1 PctA, *Bacillus subtilis* sensor histidine kinase KinD, *Rhodopseudomonas palustris* diguanylate cyclase rpHK1S-Z16, *Pseudomonas aeruginosa* chemoreceptor TlpQ) with ClustalW embedded in MEGA X. The phylogenetic tree was built using the maximum likelihood statistical method with 1000 bootstrap replications and 95% credible intervals [31] and visualized using iTOL [33].

### AI-2-related gene expression within a rumen microbial metatranscriptome

Meta-transcriptomic data was downloaded from the SRA database of the National Center for Biotechnology Information using the accession number SRA075938, which contains 20 different samples [24]. All samples were sequenced paired-end using the HiSeq2000 sequencer with a read length of 150 bp [24]. The raw data is trimmed to 110 bp by removing both the leading and trailing 20 bp ends of each read with Trimmomatic v0.39 [34] after quality checked with fastqc v0.11.9 [35] and multiqc v1.9 [36] toolsets. After trimming, reads were aligned to rumen bacterial genomes by bowtie2 v2.3.5.1 [37], and generated SAM formatted alignment files were converted to BAM format using SAMtools v1.10 [38] in order to be counted by FeatureCounts v2.0.0 [39]. Finally, these counts are calculated to form a table of RPKM values where statistics of genes of interest were extracted.

### Microbial social network based on quorum sensing in the rumen

We integrated the AI-2 signaling molecule and its associated microbes to construct the microbial social networks based on QS, which were visualized using Cytoscape software [40]. Specifically, the potential microbes containing AI-2 synthetase and/or receptors were arranged into microbial points, the predicted distinct types of AI-2 receptors were arranged into groups, which were made into Excel spreadsheets. These Excel spreadsheets were imported into Cytoscape software to construct a microbes-signal molecule-action pathways network based on the AI-2 signaling pathways. The network contains microbes, signal molecules, and action pathways. The edge associations represent the possible relationship [41]. The major microbial phyla and QS-related proteins were labeled in the network of the rumen microbial ecosystem.

## Results

### Prevalence of quorum sensing-related genes in rumen microbes

**Fig. 1:**
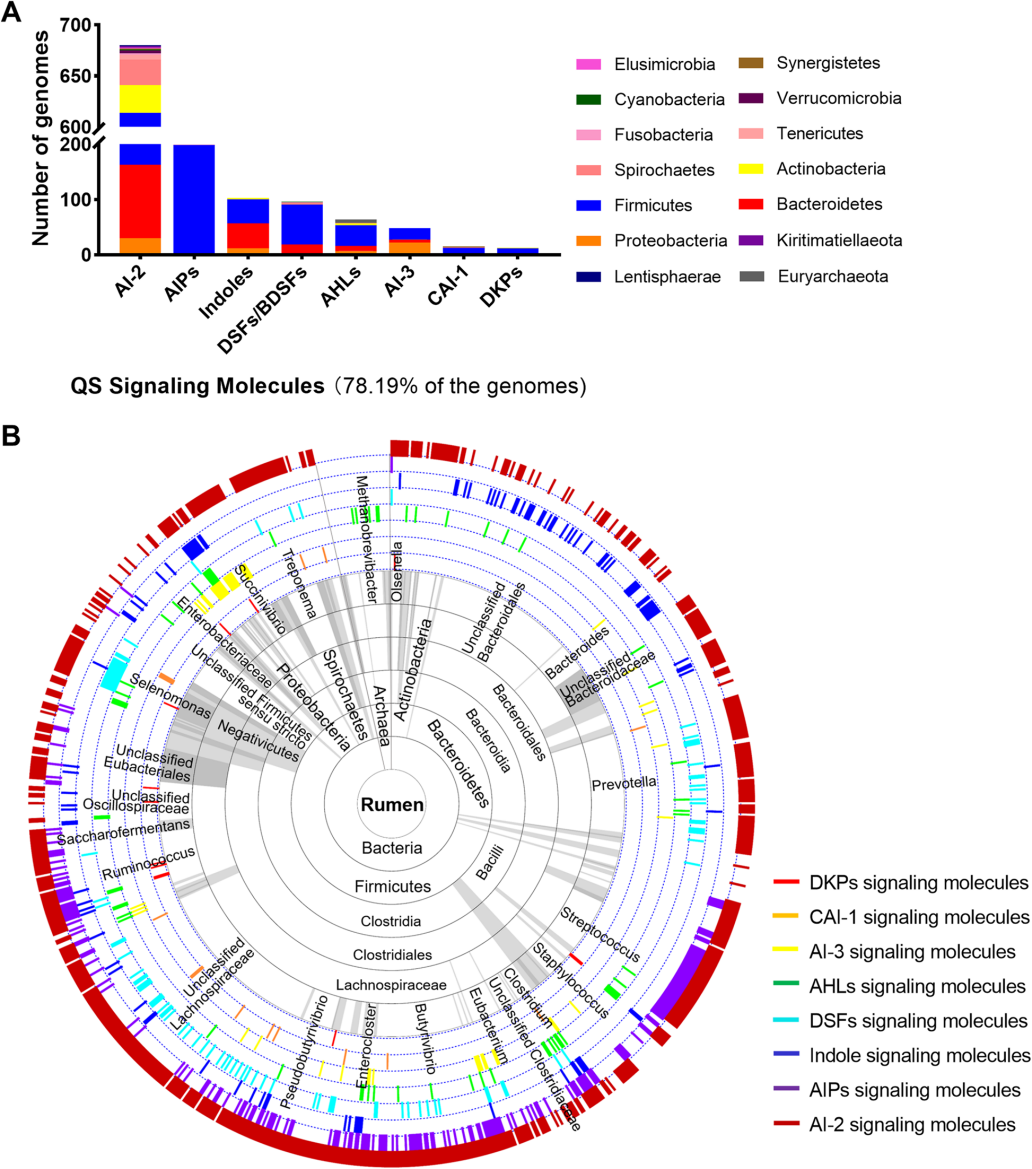
Prevalence of putative QS molecule-related proteins within rumen microbial genomes. **a** Prevalence of putative QS molecule-related proteins in rumen microbial genomes. **b** Microbial taxonomic ranks and distribution of QS molecule-related proteins in rumen microbes. Different colored blocks (white, gray, and dark gray) in the inner cycle indicate microbial taxa. Flags in the outer eight layers indicate the QS molecule-related proteins present in the corresponding genome

Ruminal microorganisms are critical for the feed conversion efficiency and energetic efficiency of ruminants [42, 43]. To study the communication of rumen microbes based on QS in these microbial processes, 1045 rumen microbial genomes were obtained from the Joint Genome Institute (JGI) database and National Center for Biotechnology Information (NCBI) database, and 981 bacterial and archaeal genomes were retained based on the criteria of ≥80% completeness and ≤10% contamination according to CheckM, among which 948 were bacterial genomes and 33 were archaeal genomes (Table S2). We searched for homologs of all 15 types of known QS signaling molecule-related genes and domains and found 8 homologs distributed in 767 (out of 981, 78.19%) of the rumen microbial genomes, including 761 bacterial and 6 archaeal genomes. Specifically, only 13 (1.33%) of the 981 microbial genomes analyzed possessed dimodular nonribosomal peptide synthases (DKP-related proteins) that can produce pyrazinone metabolites [44]; 15 (1.53%) species contained the autoinducer sensor kinase/phosphatase CqsS, which is membrane-bound protein that can sense CAI-1 [45]; 48 (4.89%) and 96 (9.79%) species contained genes encoding the QseB/QseC (AI-3) and RpfC/RpfG (DSFs) two-component systems, respectively, which may be involved in signal perception and transduction [15, 46]; 103 bacterial species employed indoles, among which 79 species contained *tna*A, encoding a tryptophanase that generates indole, and 30 species contained genes encoding sensor kinases (BaeS and CpxA) and the transcriptional regulator GadX [9, 47]; 199 (20.29%) species contained *agr*A/*agr*B genes, which may participate in regulating a two-component transcriptional quorum-sensing system by sensing AIPs; 58 bacterial species appeared to contain AHL degradation genes and/or AHL regulator genes, two of which also exhibited AHL synthase genes, and 6 archaeal species only exhibited AHL degradation genes encoding *N*-acyl homoserine lactonase; and 680 (69.32%) species contained genes encoding *lux*S synthases and *luxP*, *lsrB*, and *cahR* receptor genes and might utilize AI-2 in the rumen (Fig. 1a and Table S2). Among these taxa, the largest numbers of AI-3- and AHL-related genes were observed in the phylum Proteobacteria, DSFs/BDSFs were mainly found in the class Negativicutes, and almost all of the AIP-related genes were found in the phylum Firmicutes. Indole-related genes were largely detected in the phylum Bacteroidetes, while AI-2-related genes were widespread throughout the rumen bacteria (Fig. 1b and Table S2). To further assess the prevalence of QS signaling molecules in rumen microbes, we analyzed the presence of their related genes in other rumen ecosystems (including those of cattle, sheep, moose, deer, and bison) and the pig gut ecosystem. Similar types of QS signaling molecules were observed in the rumen and pig gut, but they were more numerous in the rumen than in the pig gut. Specifically, 78.19% (Table S2 and Fig. 1a) and 67.64% (Table S3 and Figure S1a) of the rumen microbial genomes in the two rumen databases from different sources were observed to contain QS signaling molecules, whereas only 53.01% of the genomes from pig intestines contained signaling molecules (Table S4 and Figure S1b). These results demonstrated the prevalence of quorum sensing-related genes in rumen microbial genomes, suggesting that the QS system may be very important for rumen microbial communication.

### Occurrence of AI-2 synthases and receptors in rumen bacteria

**Fig. 2:**
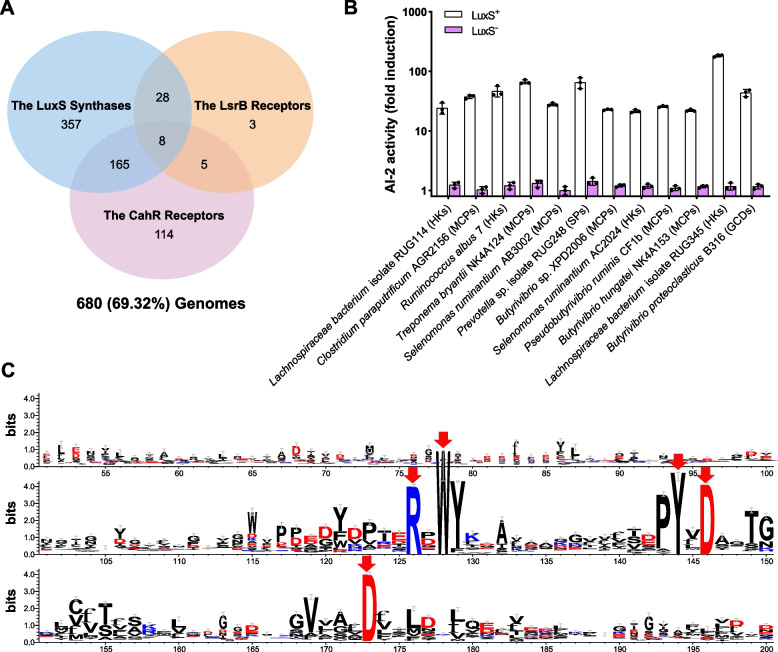
Occurrence of putative AI-2 synthase and receptor-based QS in rumen bacterial genomes. **a** Occurrence of the putative LuxS synthases, LsrB, and CahR-type receptors. Numbers indicate the numbers of bacterial genomes in which the corresponding proteins were found. Putative LuxS synthases, LuxP receptors, LsrB receptors, and CahR-type receptors were observed in 558, 0, 44, 292 rumen bacterial genomes, respectively. **b** The dCache_1 domains from the CahR-type AI-2 receptors predicted to function as MCPs, HKs, GCDs, and SPs in rumen bacteria are capable of retaining AI-2. **c** Model of the conservation of the dCache_1 domain. Sequence logo of a multiple sequence alignment of 638 putative dCache_1 domain sequences of CahR-type AI-2 receptors with the five conserved positions (R126, W128, Y144, D146, and D173) of *P. aeruginosa* PctA in rumen microbes. Multiple alignment analysis was performed with ClustalW in MEGA X. The logo was visualized with WebLogo 3

**Fig. 3:**
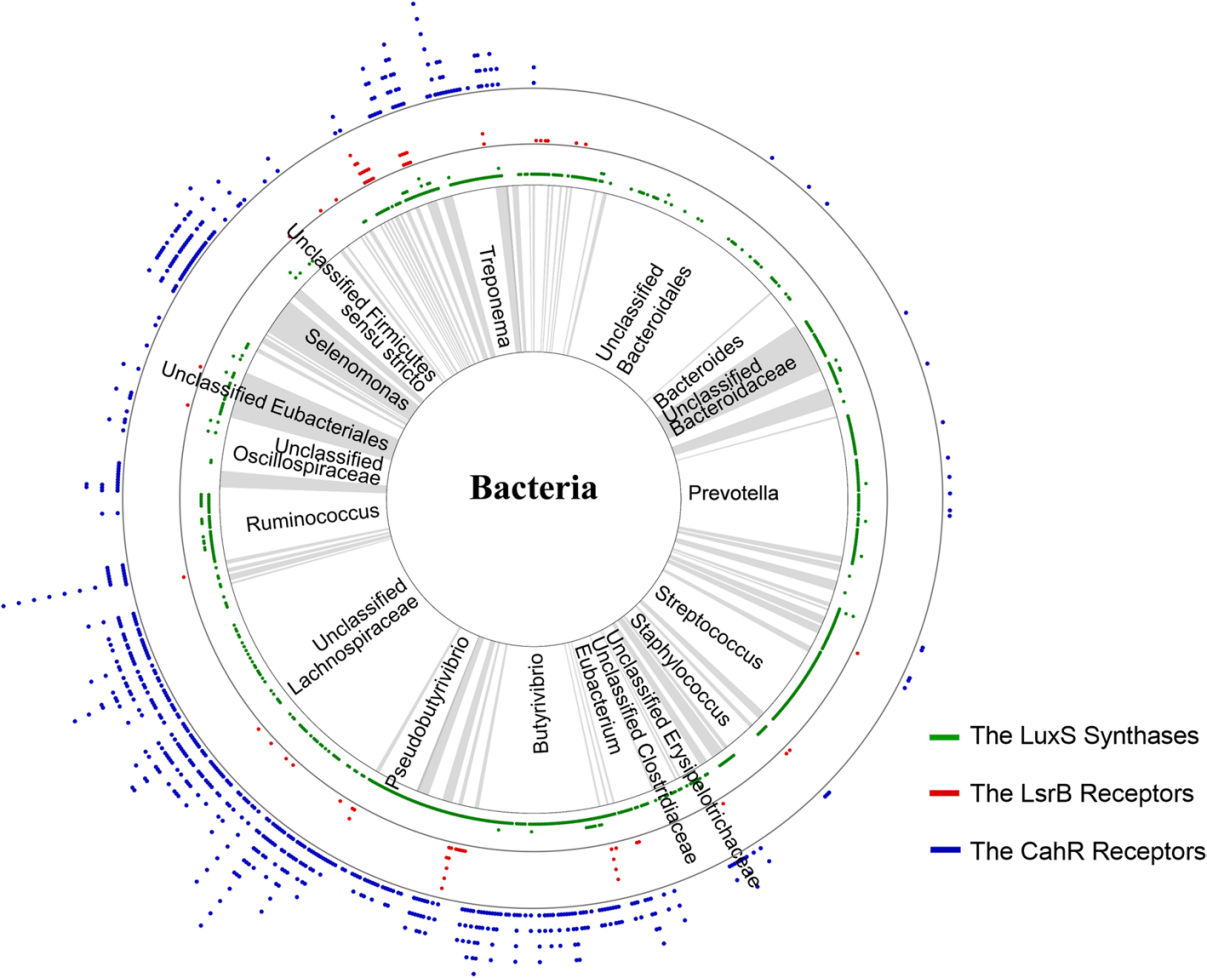
The distribution and abundance of AI-2 synthase- and receptor-based QS in the rumen bacterial genomes. Different colored blocks (white, gray, and dark gray) in the inner cycle indicate bacterial taxa. Points in the outer three layers indicate the putative LuxS synthases, LsrB receptors, and CahR-type receptors present in the corresponding genome. The points represent the abundance of AI-2 synthases or receptors. Green points represent putative LuxS synthases, red points represent putative LsrB receptors, and blue points represent putative CahR-type receptors

AI-2 could be used as an important type of “bacterial Esperanto” for interspecies communication, which has been recognized as a major determinant of bacterial group behaviors. We performed further detailed analyses of the key genes involved in the LuxS/AI-2 QS-related pathway of rumen microbes. The *luxS* gene is responsible for the biosynthesis of AI-2. A total of 609 LuxS proteins were identified in 558 genomes (Fig. 2a and Table S2). The known receptors LuxP and LsrB, which can respond to AI-2 to modulate the expression of multiple genes in the bacterial kingdom [4, 22, 48], were observed in 0 and 44 rumen bacterial genomes, respectively (Fig. 2a and Table S2). The dCACHE domain acts as an extracytoplasmic sensor of various signal transduction proteins in prokaryotes, and the dCache_1 domain subfamily is the largest subfamily of the dCACHE family [32, 49]. Our previous work revealed that novel CahR type AI-2 receptors containing dCache_1 with five conserved amino acid residues, corresponding to R126, W128, Y144, D146, and D173 in the active pocket of PctA-LBD (ligand-binding domain) of *P. aeruginosa*, mediate communication in prokaryotes [22]. We thus identified 4041 dCache_1 domains in 656 genomes from both bacteria and archaea, including 638 CahR-type receptors in 292 bacterial genomes (Fig. 2a). To evaluate the ability of these CahR receptors to bind extracellular AI-2 in vitro, we randomly selected 12 CahR-type receptor sequences and examined the bioluminescence of *V. harveyi* strain MM32 (LuxN^-^, LuxS^-^) based on AI-2 released from purified recombinant proteins from the *luxS*^+^ *E*. *coli* strain. AI-2 binding activity was observed in all 12 proteins (Fig. 2b). Thus, the dCache_1 domains with the five conserved amino acid residues as novel AI-2 receptors are subjected to analyses hereafter (Fig. 2c). We reviewed the overall distribution of AI-2-related proteins in the rumen bacterial genomes and observed that LuxS synthases and CahR receptors were present in 59% and 31% of the rumen bacterial genomes, respectively; these proteins were highly abundant in *Prevotella*, *Ruminococcus*, *Treponerna*, and the family Lachnospiraceae (accounting for 23.85% of the total microbial genomes). *Streptococcus* contained only LuxS synthases, and *Selenomonas* contained CahR receptors (Fig. 3 and Figure S2). Most of these genera are well known to be dominant among rumen microbes and to play important roles in rumen homeostasis [50–52]. Furthermore, to gain insight into the AI-2 signaling molecules of rumen bacteria, we compared the abundance of the corresponding synthases and receptors in different ecosystems. We found that in the two rumen microbial genomic databases from different sources, 69.32% (Table S2 and Fig. 2a) and 57.03% (Table S3 and Figure S3a) of the genomes, respectively, contained AI-2 signaling molecules, whereas only 39.50% of the genomes from pig intestines contained these molecules (Table S4 and Figure S3b). Additionally, the number of AI-2 receptors in rumen microbial genomes was greater than that in pig gut genomes. A total of 32.93% (Table S2 and Fig. 2a) and 17.59% (Table S3 and Figure S3a) of the rumen microbial genomes in the two rumen databases contained AI-2 receptors, whereas only 6.53% (Table S4 and Figure S3b) of the pig gut genomes contained these receptors. These results suggest that most rumen bacteria likely employ AI-2 for QS functions and indicate the importance of the LuxS/AI-2-based QS system in this ecosystem.

### Diversity of CahR-type AI-2 receptors detected in rumen bacterial genomes

**Fig. 4:**
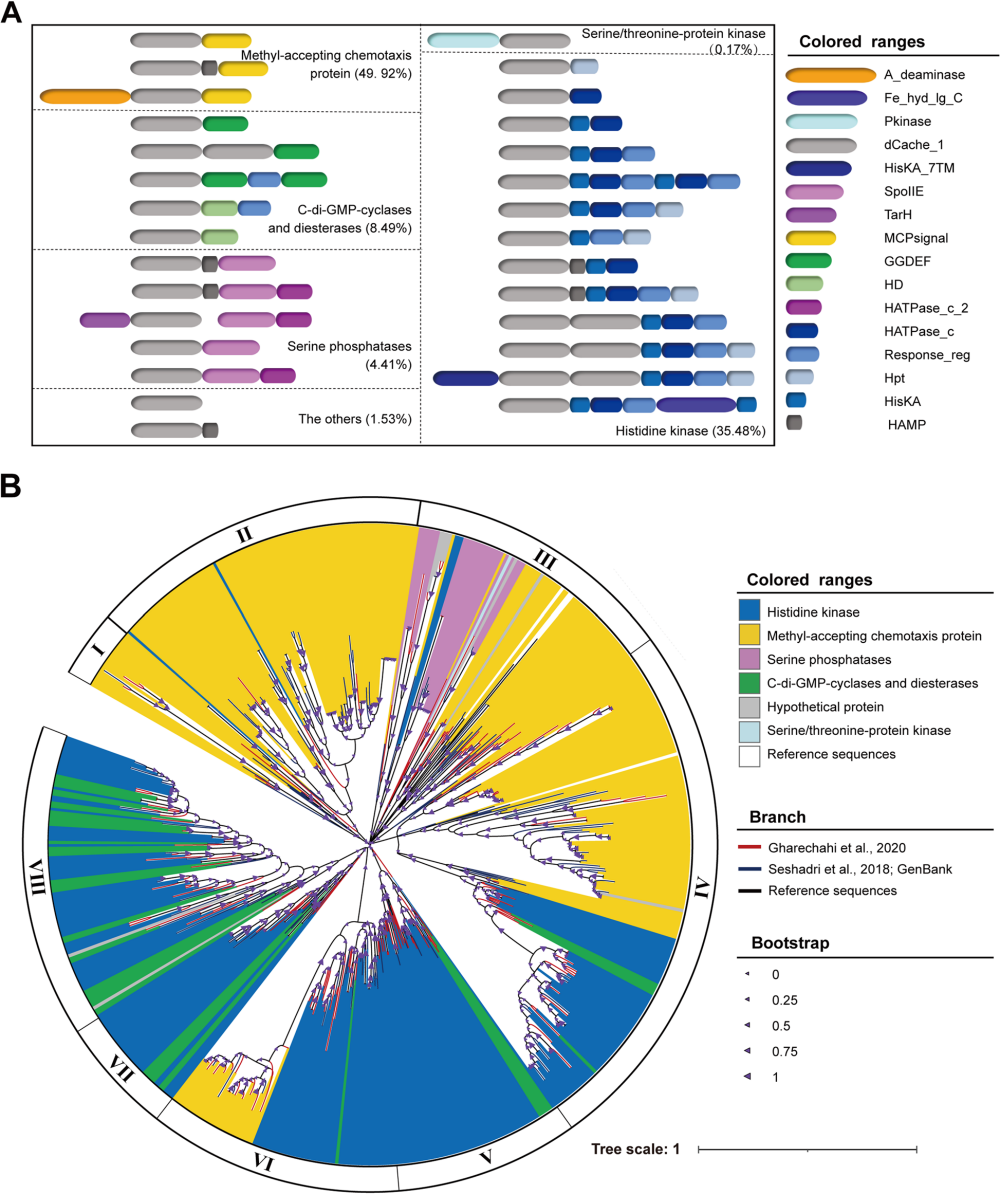
The diversity of all putative CahR-type receptors detected within 292 rumen bacterial genomes. **a** The domain architecture of 589 CahR-type receptor proteins. Domains are defined by the Pfam models with significant Pfam-A Matches and shown in colors. **b** The phylogenetic tree of 638 dCache_1 domains from 589 CahR-type receptor proteins. The tree was built based on the multiple sequence alignments with ClustalW embedded in MEGA X and visualized with iTOL. Four standards (*Pseudomonas aeruginosa* PAO1 PctA, *Bacillus subtilis* sensor histidine kinase KinD, *Rhodopseudomonas palustris* diguanylate cyclase rpHK1S-Z16, *Pseudomonas aeruginosa* chemoreceptor TlpQ) were used for comparison with other dCache_1 domains. Six groups were highlighted with colors (methyl-accepting chemotaxis protein, histidine kinase, serine phosphatases, C-di-GMP-cyclases and diesterases, serine/threonine-protein kinase, and hypothetical protein). The corresponding color range of labels represents protein types. Three colors of branches show that sequences are from the Gharechahi et al., Seshadri et al./NCBI database, and reference sequences, respectively

Compared with two known AI-2 receptors, LuxP and LsrB, CahR-type receptors are both more numerous and more widely distributed in rumen bacteria. Thus, the putative functional modules of CahR-type AI-2 receptors were explored. Among 589 putative CahR receptor proteins, 580 could potentially function as extracytoplasmic sensors of transmembrane signal transduction proteins such as MCPs (49.92%), HKs (35.48%), SPs (4.41%), GCDs (8.49%), and STKs (0.17%) (Fig. 4a and Table S5). MCPs and HKs were two of the dominant types of signal transduction proteins acting as AI-2 receptors in the rumen bacteria (Fig. 4a and Figure S4). Therefore, different QS signaling pathways may be activated following AI-2 binding to different receptors to regulate social phenotypes. Additionally, a phylogenetic tree including 638 dCache_1 domain sequences from the 589 putative CahR-type receptor proteins and four reference sequences was built, and their evolutionary relationships were visualized. According to this phylogenetic tree, the CahR-type receptors within the cultured bacterial genomes (blue branch) clustered similarly into eight separate clades in accord with rumen metagenomics datasets (red branch). Most dCache_1 domains from the same type of proteins generally clustered together, although the clustering of multiple dCache_1 domains can occur within the same type of proteins. dCache_1 domains from methyl-accepting chemotaxis proteins formed at least five phylogenetically distinct clusters (Fig. 4b), which may indicate the diversity of CahR-type receptors allowing adaptation to diverse environmental conditions in rumen. These results revealed the diversity and complexity of CahR-type receptors as potential “listen” signals and indicate the importance of CahR-type AI-2 receptors for mediating downstream signaling pathways in this QS system in the rumen.

### Expression of the identified AI-2 synthases and receptors in rumen metatranscriptome datasets

**Fig. 5:**
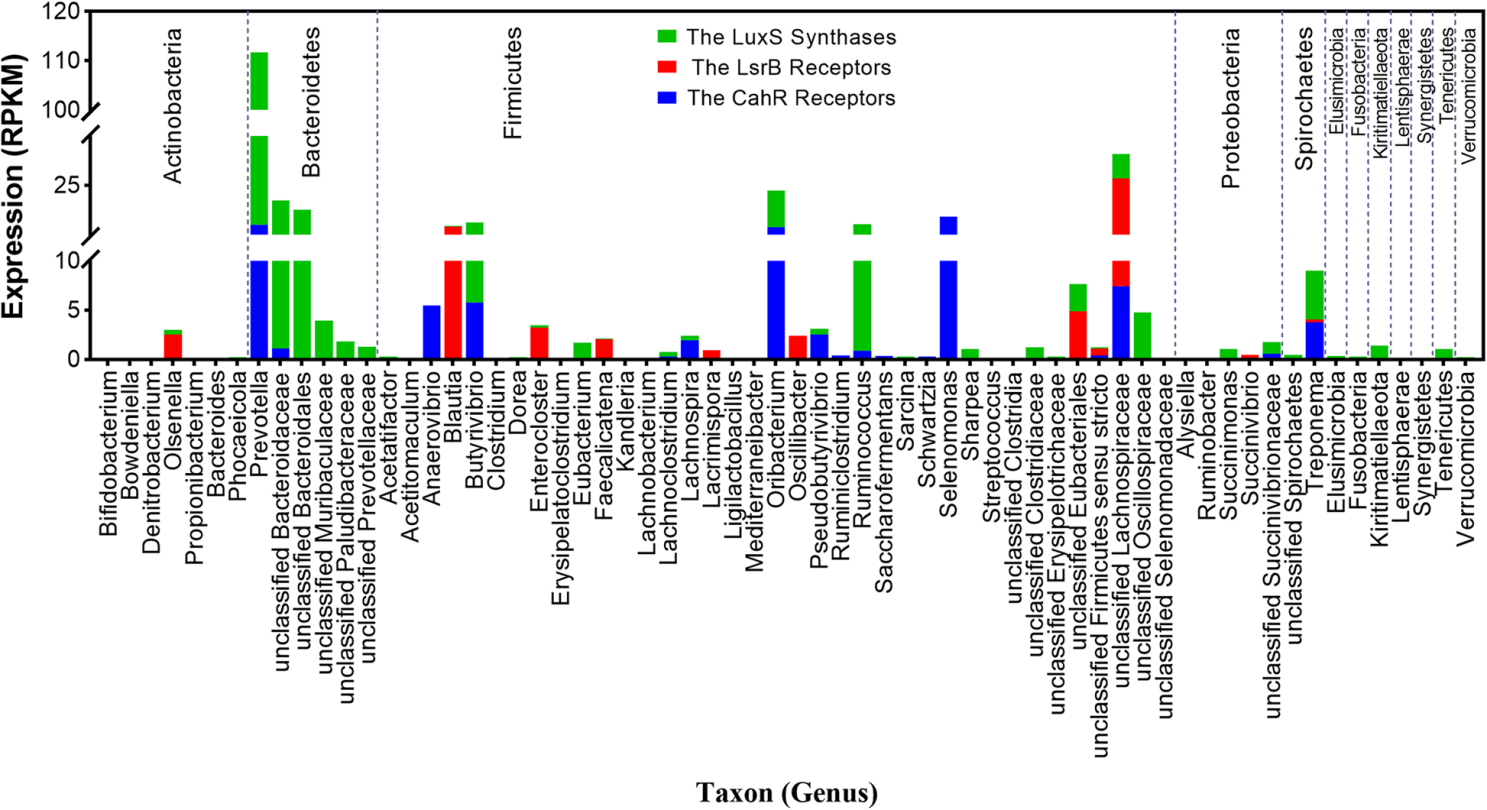
Expression of the identified AI-2 synthases and receptors within a rumen microbial metatranscriptome. The expression of the *luxS* synthase genes, *lsrB* receptor genes, and CahR-type receptor genes were discovered within 53, 12, and 36 genera, respectively

To probe the actual expression levels of AI-2 synthases and receptors within rumen bacterial genomes in vivo, we investigated their expression levels in the metatranscriptome datasets of rumen microbes (105 GB sequencing data) [24]. A total of 523 (out of 680, 77%) genomes expressed AI-2-related genes in these datasets. Specifically, 380 (out of 558, 68%) genomes expressed the AI-2 synthase gene *luxS* (Figure S5a), which was mainly distributed in the phyla Bacteroidetes and Firmicutes (Figure S5b), 26 (out of 44, 59%) genomes expressed the known receptor gene *lsrB* (Figure S5a), and 256 genomes (out of 292, 88%) expressed CahR-type receptor gene (Figure S5a), which were largely located in the phyla Firmicutes and Spirochaetes (Figure S5d). We further evaluated the average expression levels of metranscriptomic sequences (*n* = 20) at the metagenome level. High expression of AI-2 synthases and receptors was observed in *Prevotella*, *Oribacterium*, *Butyivibrio*, *Ruminococcus*, and *Treponema*, which contributed not only to the accumulation of extracellular AI-2 but also to the uptake of AI-2 and thus modulated bacterial communications, as observed in *Prevotella* sp. isolate RUG248 and *Oribacterium* sp. NK2B42. *Selenomonas* expressed only CahR-type receptors mediating the uptake of AI-2 from the extracellular environment; for example, high expression levels were observed for CahR-type receptors originally detected within *Selenomonas ruminantium* ATCC 12561 and *Selenomonas ruminantium* L14 (Fig. 5 and Table S6). In general, expression levels may differ under different microecological conditions, as metatranscriptomic datasets record only a transient snapshot at a single point in time under certain environmental conditions, which means that the expression of originally identified genes that were not observed within the metatranscriptome datasets may be detected in different microecological environments. Overall, the high expression levels of AI-2 synthases and receptors identified within the rumen microbes confirmed that rumen bacteria mainly talked with each other through AI-2 signaling molecules.

### AI-2-based quorum sensing mediates widespread interspecies communications within rumen bacterial communities

**Fig. 6:**
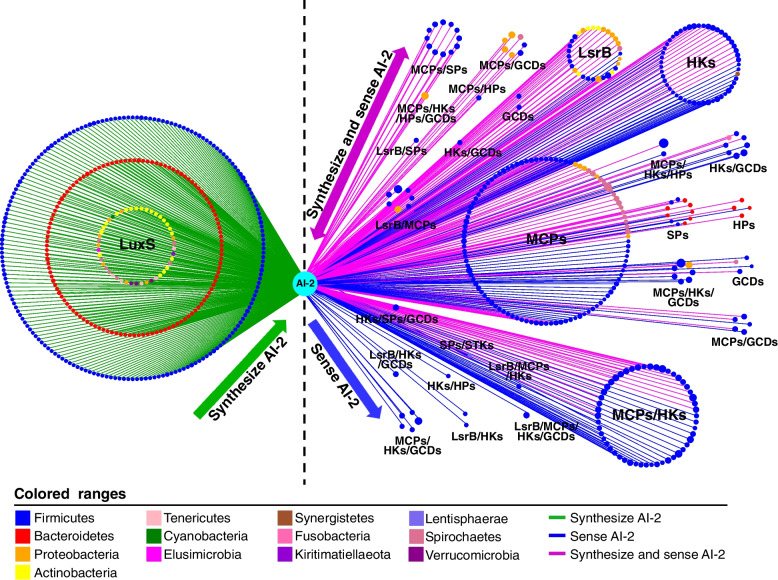
AI-2-based QS-mediated bacterial communication network in the rumen. The nodes represent microbial species and AI-2 signaling molecules. The colors of the nodes represent bacterial phyla and AI-2 signaling molecules. Green edges indicate that the bacterial species possessing LuxS synthases produce AI-2 signaling molecules, blue edges indicate that AI-2 binds to the corresponding receptors, and purple edges indicate that the bacterial species possessing both LuxS synthases and receptors can produce and sense AI-2 signaling molecules

To better understand the communications across rumen microbes, we focused on the sophisticated QS networks between AI-2 signaling molecules and bacterial species containing AI-2 synthases and/or receptors based on QS signaling pathways. The nodes in the network represent rumen microbes and AI-2 signaling molecules, and the edges represent possible communications (green edges represent that the bacterial species containing *luxS* synthases produce AI-2, blue edges represent AI-2 binds to the corresponding receptors in bacteria, purple edges indicate that the bacterial species possessing both LuxS synthases and receptors can produce and sense AI-2). Most communications were universal in the bacteria containing AI-2 synthase LuxS and/or CahR receptors. A total of 357 bacterial species containing only AI-2 synthases were responsible for the production of the AI-2 signaling molecules; 122 containing only receptors (including 3 possess the known receptors LsrB, 114 possess the CahR receptors, and 5 possess both LsrB and CahR receptors) sensed AI-2 to reprogram the expression of multiple genes; 201 containing both synthetases and receptors not only produced AI-2 but also sensed AI-2 in the environment to activate proteins such as MCPs, HKs, SPs, GCPs, and STKs to perform social functions (Fig. 6 and Figure S6). MCPs, as dominant AI-2 receptor proteins, mainly function in Firmicutes, Spirochaetes, and Proteobacteria (Figure S7a), and HKs function in Firmicutes, Bacteroidetes, and Proteobacteria (Figure S7b). In total, 204 bacteria contained AI-2 receptor MCPs, and 133 contained HKs. Sixty-eight bacterial species contained more than two types of receptor proteins, such as *Succinivibrionaceae bacterium* isolate RUG84, which contained not only MCPs but also HKs, GCDs, and HPs. *Lachnospiraceae bacterium* isolate RUG371 contained HKs, SPs, and GCDs (Fig. 6 and Table S7). As the contribution of each QS pathway to total gene expression may be distinct, it is essential to understand that the microbes regulate group behaviors via the QS network in the rumen. The complex signaling network with multiple input points likely plays a pivotal role in regulating microbial group behavior and may be conducive to revealing the key relationships between microbes and AI-2 signaling molecules. These results highlight the importance of AI-2 in QS networks and indicate that AI-2 cross-talk with global regulatory networks mediates widespread interspecies communication within rumen bacterial communities.

## Discussion

Quorum sensing is a microbial cell–cell communication process that allows microbes to function as a collective group. However, due to the complexities of the rumen ecosystem, how these microbes interact and work together to degrade plant feed in the rumen is still unclear. Here, we explore a novel way to solve this problem by building a communication network between QS signaling molecules and microbes in the rumen. In this network, AI-2 signaling molecules are produced and secreted into the environment by AI-2-producing bacteria, and these signaling molecules can be sensed by AI-2 receptor-containing bacteria to modify group behaviors by activating corresponding genes, such as those encoding MCPs, HKs, SPs, GCDs, and STKs, which have diverse functions and are involved in different signaling pathways. Our analysis contributes to understanding complex microbial communication networks in the context of QS and how AI-2 functions as a signal for bacterial communication in rumen ecosystems despite some inherent limitations of large-scale network analysis [53].

Most current research is focused on cell communications with a nonruminant origin and communications in monospecies cultures of species such as *E. coli*, *P. aeruginosa*, *V. harveyi*, *Streptococcus gordonii*, and *Staphylococcus aureus* [54–58]. With the development of genomic and microbiological technologies, it has become possible to assess the impacts of QS signaling molecules on complex microbial communities in the rumen ecosystem. In our study, we explored the distribution and abundance of genes related to 15 types of QS signaling molecules in rumen microbial genomes collected from the Hungate collection [24] and GenBank [25], which represent many of the available rumen microorganisms and rumen microbial metagenomes. Our results suggest that most rumen microbes may employ AI-2 as a QS signaling molecule to regulate social behaviors in this ecosystem.

AI-2 is a universal QS signal and is therefore likely to be an important signal in microbiomes or natural consortia [59]. The *luxS* gene is responsible for the biosynthesis of AI-2 and is widespread throughout the bacterial kingdom [48]. Most studies of QS in microecosystems have focused on the distribution and abundance of the *luxS* gene and its homologs [18–21, 60, 61]. However, QS requires not only a synthase to send a signal but also a receptor to sense the signal, and few studies have investigated the receptors required for QS. The inability to identify AI-2 sensors in AI-2-responsive microbes has greatly hampered our understanding of the role of AI-2 as a universal QS signaling molecule participating in intra- and interspecies communications. Therefore, this study was conducted not only to investigate the *luxS* gene but also to perform an in-depth analysis of the corresponding receptors. Approximately 59% of our reference rumen bacterial genomes contained *luxS* genes, which were mainly distributed in bacterial genomes and were widespread. Two previously identified receptors, LuxP and LsrB, are members of the periplasmic binding protein (PBP) family that contain periplasmic sensory domains that can transmit AI-2 signals into cells by employing the corresponding transmembrane signaling pathways [62, 63], but they were only present in a small number of genomes (4.5%). Previous studies have provided evidence that LuxP is only present in Vibrionales and that LsrB occurs in members of the Rhizobiaceae and Bacillaceae families, and other enteric bacteria [23, 63, 64]. Few of these taxa were included in our rumen microbial genomic database, which led us to focus on exploring new receptors. Cache domain proteins are the most abundant extracellular sensors in prokaryotes [32] and appear to respond to a range of different types of ligands [22, 65]. The discovery of dCache_1-containing proteins and their role in AI-2 sensing has expanded our knowledge of AI-2 receptors. These CahR-type AI-2 receptors are transmembrane proteins with dCache_1 domains [22] and are present in 31% of the rumen microbial genomes. Therefore, our results not only confirm that the LuxS proteins may be employed by bacteria as previously suggested [21, 48, 66] but also reveal the new finding that many bacteria can sense AI-2 signals via receptor proteins harboring dCache_1 domain sensors in the rumen.

dCache_1 domains are present in all major transmembrane signal transduction proteins in prokaryotes, including MCPs (MCP signal), HKs (HisKA, HisKA_2, HisKA_3, HWE_HK, HATPase_c, HATPase_c_2, HATPase_c_3, HATPase_c_5), GCDs (GGDEF, EAL, HD-GYP), SPs (SpoIIE, PP2C, PP2C_2), ACs/GCs (guanylate_cyc), and STKs (Pkinase) [32]. In our study, we observed that MCPs and HKs were the dominant types of signal transduction proteins among all AI-2 receptors of rumen bacteria. Therefore, they may serve as core signal transduction proteins in the rumen ecosystem. MCPs are the most common chemotaxis receptors in prokaryotes [67], and they can sense chemical cues and transmit signals to cytoplasmic pathways that are responsible for chemotaxis behaviors [68]. For example, in *E. coli*, this type of signaling inhibits the change in the rotational direction of flagella from counterclockwise to clockwise to maintain smooth swimming, leading to chemotaxis [67]. Multiple studies have demonstrated that chemotaxis strongly affects the cell adhesion of *E. coli*, which might in turn affect the initial colonization of a abiotic surface [69] during biofilm formation [70]. These cellular activities have also been observed in other bacteria [71–73]. In our research, we speculated that the dCache_1 domains included MCPs can sense environmental AI-2 and then transfer the AI-2 signal to the cytoplasmic signaling domain, which may modulate chemotaxis toward AI-2 released by other microbes, thus underlying the ability of bacteria to colonize plant feed and promote biofilm formation. Within biofilms, a consortium of microbes is encased in a self-produced extracellular capsule, and digestive enzymes are concentrated in proximity to the substrate, an arrangement that enables the effective hydrolysis of plant feedstuffs within the rumen [2].

HKs, which are another dominant type of signaling protein associated with dCache_1, are also crucial in rumen bacterial activities. HKs comprise a broad range of proteins with a wide variety of functions, and some of these functions are very similar to those of MCPs. For example, previous studies have suggested that the histidine kinase CheA is the core chemotactic signaling and phosphorylation modulator in the rotation of flagella to reorient the cell swimming direction [69, 74]. Additionally, another kind of HK, RcsC, serves as the initiating kinase in some phosphorylation cascades and is essential in Rcs phosphorelay [75] signaling to regulate swarming behavior and extracellular matrix production in *E. coli* [76]. HKs can also participate in some important biological processes other than chemotaxis; for example, KinD is involved in sporulation and biofilm formation in *Bacillus subtilis* [77]. More importantly, HKs can regulate citrate and malate metabolism in the rumen microbial ecosystem by responding to C4-dicarboxylates or citrate, which stimulate fermentation in the rumen [17]. Overall, dCache_1-containing HKs may play a role in events including chemotaxis toward AI-2 released by other microbes, adhesion, and the colonization of the plant feed surface to form biofilms via capsule production, ultimately contributing to rumen fermentation to provide energy and nutrients to the host. In this model, the dCache_1 domains of the HKs sense environmental AI-2 and transmit the provided information to the cytoplasmic kinase domain to trigger the phosphorylation cascade in downstream signaling events [78]. Therefore, we conclude that the QS processes mediated by CahR-type AI-2 receptors may be essential for degrading plant material, digesting food, and providing energy and nutrients to the host.

Previous studies that investigated the presence of *luxS* genes and their homologs in rumen genetic materials suggested that *Prevotella*, *Butyrivibrio*, *Streptococcus*, *Ruminococcus*, *Treponema*, and *Pseudobutyrivibrio* are the dominant genera in the rumen ecosystem and may possess AI-2 QS signaling ability [18, 20, 60]. In our study, we illustrated that most of these bacteria, including *Prevotella*, *Butyrivibrio*, *Ruminococcus*, *Clostridium*, *Selenomonas*, *Treponema*, and *Pseudobutyrivibrio*, were dominant and occupied crucial ecological niches in the rumen [79, 80], probably owing to AI-2 receptors such as MCP and HKs that contribute to the successful attachment and colonization of the dominant bacteria. Most of the AI-2 synthases and receptors showing high expression in the rumen metatranscriptome datasets support the idea that QS is widespread among rumen microbes and that most rumen bacteria have the capacity to mediate both intraspecies and interspecies communications via AI-2-based QS. In addition, 178 of the 558 identified *luxS* genes, 18 of the 44 identified *lsrB* genes, and 36 of the 292 identified the CahR receptor genes showed no detectable expression in the metatranscriptome datasets; however, AI-2-binding activity was observed in 12 proteins in vitro, including three proteins that were not expressed in these datasets. The reason for the lack of expression in the rumen was unclear, but it was presumably a result of gene silencing and transient gene expression under specific conditions.

## Conclusion

We identified AI-2 synthases and multiple types of receptors in rumen microbes via the major genomic resources available and constructed a QS regulatory network of AI-2 signaling, in which AI-2 was produced and bound to receptors to regulate microbial group functions. We have in-depth knowledge about how bacteria utilize AI-2-based QS to communicate with each other and coordinate social behaviors in the rumen. To our knowledge, this is the first study to report the distribution, abundance, evolutionary relationships, and potential functions of the dCache_1 domain as an AI-2 receptor in the microecosystem. The current study thus further expands the understanding of unknown components of the QS system in the microenvironment. Further studies should be focused on the possible synergistic interaction mechanisms and implications of rumen microorganism-based QS in plant degradation, energy utilization, and disease control.

## Supplementary Information

**Additional file 1: Table S1.** Quorum Sensing molecules and their related genes.

**Additional file 2: Table S2.** The rumen microbiota and the presence of quorum sensing molecules.

**Additional file 3: Table S3.** The rumen microbiota and the presence of quorum sensing molecules in the rumen ecosystems of species including cattle, sheep, moose, deer, and bison (as the control group).

**Additional file 4: Table S4.** The microbes and the presence of quorum sensing molecules in the pig gut (as the negative control group).

**Additional file 5: Figure S1.** The presence of putative QS molecule-related proteins in microbial genomes from different sources. (A) Putative QS molecule-related proteins in the rumen ecosystem, including cattle, sheep, moose, deer, and bison (as the control group). (B) Putative QS molecule-related proteins in the pig gut (as the negative control group).

**Additional file 6: Figure S2.** Distribution and abundance of AI-2 synthases and receptors based on QS in the rumen bacterial genomes. Numbers indicate the number of bacterial genomes in which the corresponding proteins were found at the genus level. The LuxS synthases, LsrB receptors, and CahR-type receptors were discovered within 88, 27, and 42 genera, respectively.

**Additional file 7: Figure S3.** Occurrence of putative AI-2 synthase- and receptor-based QS in microbial genomes from different sources. (A) Putative LuxS synthases, LsrB, and CahR-type receptors in the rumen ecosystems including cattle, sheep, moose, deer, and bison (as the control group). (B) Putative LuxS synthases, LsrB, and CahR-type receptors in the pig gut (as the negative control group). Numbers indicate the numbers of bacterial genomes in which the corresponding proteins were found.

**Additional file 8: Table S5.** The diversity of predicted CahR-type receptors.

**Additional file 9: Figure S4.** The types of the CahR-type receptors. Numbers indicate the number of bacterial genomes in which the corresponding proteins were found. The MCPs and HKs were two dominant AI-2 receptor proteins in rumen bacteria.

**Additional file 10: Table S6.** Expression of the identified AI-2 synthases and receptors in rumen metatranscriptome datasets.

**Additional file 11: Figure S5.** Expression of the predicted AI-2 synthases and receptors within a rumen microbial metatranscriptome at the phylum level. (A) The number of bacterial genomes containing AI-2-related genes and the percentage of corresponding genomes that expressed these genes. (B) The number of bacterial genomes containing *luxS* synthases and the percentage of the corresponding genomes that expressed *luxS* genes at the phylum level. (C) The number of bacterial genomes containing *lsrB* receptors and the percentage of corresponding genomes that expressed *lsrB* genes at the phylum level. (D) The number of bacterial genomes containing CahR-type receptor genes and the percentage of the corresponding genomes that expressed these genes at the phylum level. Numbers indicate the numbers of bacterial genomes in which the corresponding functions were found at the phylum level. The phyla (> 0.6% of total microbial genomes) were selected. A total of 380 (68% of 558,) genomes expressed *luxS* synthases, which were mainly distributed in the phyla Bacteroidetes and Firmicutes; 26 (59% of the 44) genomes expressed known receptor *lsrB* genes; and 256 genomes (88% of 292) expressed CahR-type receptors, which were largely located in the phyla Firmicutes and Spirochaetes.

**Additional file 12: Figure S6.** Widespread occurrence of rumen bacteria in the communication network. (A) The occurrence of rumen bacteria in the network. (B) The occurrence of bacteria that synthesize AI-2 at the phylum level. (C) The occurrence of bacteria that synthesize and sense AI-2 at the phylum level. (D) The occurrence of bacteria that sense AI-2 at the phylum level. Numbers indicate the numbers of bacterial genomes in which the corresponding functions were found at the phylum level. A total of 357 bacterial species were responsible for the synthesis of AI-2, which were mainly distributed in Actinobacteria, Bacteroidetes, and Firmicutes; 122 bacterial species sensed AI-2 to reprogram the expression of multiple genes, which were mainly distributed in Firmicutes; 201 bacterial species could not only produce AI-2 but also sense AI-2 in the environment, which primarily functioned in Firmicutes.

**Additional file 13: Figure S7.** Widespread occurrence of MCPs and HKs as dominant AI-2 receptors in communication networks.

**Additional file 14: Table S7.** The network among the rumen bacterial taxa and AI-2.

## Data Availability

The 538 and 2809 rumen microbial metagenome assemblies analyzed in this study are available at the NCBI BioProject with the accession number PRJNA631951 and 10.6084/m9.figshare.12164250, respectively. The 6339 pig gut microbial metagenome assemblies are available at the China National GeneBank DataBase (CNGBdb) with the accession number CNP0000824. Other microbial genomes are available at the IMG portal (https://img.jgi.doe.gov/) and the NCBI (https://www.ncbi.nlm.nih.gov/genome/), including 410 genomes are available (https://genome.jgi.doe.gov/portal/HungateCollection/HungateCollection.info.html). Twenty publicly available meta-transcriptomic datasets were downloaded from the SRA database of the NCBI using accession number SRA075938.

## Abbreviations

QS: Quorum sensing
AIs: Autoinducers
AHLs: N-acyl homoserine lactones
AIPs: Autoinducing peptides
AI-2: Autoinducer-2
DSFs: Diffusible signal factors
BDSFs: *Burkholderia cenocepacia* diffusible signal factors
AI-3: Autoinducter-3
CAI-1: Cholerae autoinducer-1
DKPs: Diketopiperazines
PQS: *Pseudomonas* quinolone signal
HHQ: 2-Heptyl-4-quinolone
IQS: 2-(2-hydroxyphenyl)-thiazole-4-carbaldehyde
PPYs: Pyrones
DARs: Dialkylresorcinols
CHDs: Cyclohexanediones
CSP: Competence stimulating peptide
DPO: 3,5-Dimethylpyrazin-2-ol
3-OH-PAME: 3-Hydroxypalmitic acid methyl ester
3-OH-MAME: Methyl 3-hydroxymyristate
DPD: 4,5-Dihydroxy-2,3-pentanedione
SRH: S-ribosylhomocysteine
MCPs: Methyl-accepting chemotaxis proteins
HKs: Histidine kinases
GCDs: C-di-GMP synthases and phosphodiesterases
STKs: Serine/threonine kinases
SPs: Serine phosphatases
ACs/GCs: Adenylate- or guanylate cyclases
PBPs: The periplasmic binding proteins
JGI: Joint Genome Institute
NCBI: National Center for Biotechnology Information
MAGs: Metagenome-assembled genomes
