# Supplementary material for: Exploring AI-2-mediated interspecies communications within rumen microbial communities
Source: Microbiome. 2022 Oct 7;10:167. doi: 10.1186/s40168-022-01367-z (PMC9540692; doi:10.1186/s40168-022-01367-z)
Supplement: Supplementary file 2 — Additional file 1: Table S1. Quorum Sensing molecules and their related genes. [file 40168_2022_1367_MOESM1_ESM.docx]

| Signaling molecules | Signaling molecule related genes |
| --- | --- |
| AHLs | AhyI, AinS, AlpI, AqsI, AurI, AvsI, BjaI, BmaI, BmuI, BraI, CarI, CciI, CepI, CinI, CmrI, CsaI, CviI, EanI, EsaI, ExpI, HalI, HanI, LasI, LuxI, MrhI, MrtI, NmuI, PagI, PcoI, PhzI, PpuI, PsyI, RaiI, RhiI, RhlI, RpaI, SinI, SmaI, SplI, SpnI, SprI, SwrI, TofI, VanI, VsmI, YenI, YpeI, YpsI, YruI, YspI, YtbI, HdtS, LuxM, OpaM, VanM, Homoserine lactone, N-acyl homoserine, AHL synthase, Acyl-homoserine-lactone synthase, AhyR, AinR, AlpR, AqsR, AurR, AvsR, BjaR, BmaR, BmuR, BraR, CarR, CciR, CepR, CinR, CmrR, CsaR, CviR, EanR, EsaR, ExpR, HalR, HanR, LasR, LuxR, MrhR, MrtR, NmuR, PagR, PcoR, PhzR, PpuR, PsyR, QscR, RaiR, RexR, RhiR, RhlR, RpaR, SinR, SmaR, SplR, SpnR, SprR, SwrR, TofR, TriR, TraR, VanR, VsmR, YenR, YpeR, YpsR,YruR, YspR, YtbR, LuxN, VanN, SdiA, The lactonase, AttM |
| AIPs | AgrD, AgrB, AgrC, AgrA, Accessory gene regulator protein |
| Indoles | Tryptophanase, TnaA, PykA, BaeS, CpxA, GadX, Mtr permease, Indoles |
| DSFs/BDSFs | RpfF, RpfC, RpfR, RpfG, Diffusible signaling factor, DSF, BDSF |
| AI-3 | Tdh, Threonine dehydrogenase, Abortive tRNA synthetase, Aminoacyl-tRNA synthetases, MetRS, QseC, QseB, AI-3, Autoinducer-3 |
| CAI-1 | CqsA, CqsS, CAI-1, Cholerae autoinducer-1 |
| DKPs | Nonribosomal peptide synthetases, NRPSs, Cyclodipeptide synthases, CDPSs, nonribosomal peptide synthetase, AusA |
| PQS/HHQ | PqsA, PqsB, PqsC, PqsD, PqsE, PqsH, PhnA, PhnB, PqsR, MvfR, MexG, Pseudomonas quinolone signal |
| IQS | AmbB, AmbC, AmbD, AmbE |
| PPYs | PpyS, PluR, Photopyrones, Pyrone |
| DARs/CHDs | DarA, DarB, DarC PauR, DARs, Dialkylresorcinol, CHDs |
| CSP | ComC, ComD |
| DPO | VqmA, VqmR |
| 3-OH-PAME/3-OH-MAME | PhcB, PhcS, PhcR, PAME, Hydroxyl-palmitic acid methyl ester |

Notes: AHLs, N-acyl-homoserine lactonses; AIPs, Autoinducing peptides; DSFs, Diffusible signal factors; BDSF, *Burkholderia cenocepacia* diffusible signal factors; AI-3, Autoinducter-3; CAI-1, Cholerae autoinducer-1; DKPs, Diketopiperazines; PQS, Pseudomonas quinolone signal; HHQ, 2-heptyl-4-quinolone; IQS, Hydroxyphenyl thiazole carbaldehyde; PPYs, Pyrones; DARs, Dialkylresorcinols; CHDs, Cyclohexanediones; CSP, Competence stimulating peptide; DPO, 3,5-dimethylpyrazin-2-ol; 3-OH-PAME, 3-hydroxypalmitic acid methyl ester; 3-OH-MAME, Methyl 3-hydroxymyristate.
