# Supplementary figures and images for: Exploring AI-2-mediated interspecies communications within rumen microbial communities
Source: Microbiome. 2022 Oct 7;10:167. doi: 10.1186/s40168-022-01367-z (PMC9540692; doi:10.1186/s40168-022-01367-z)

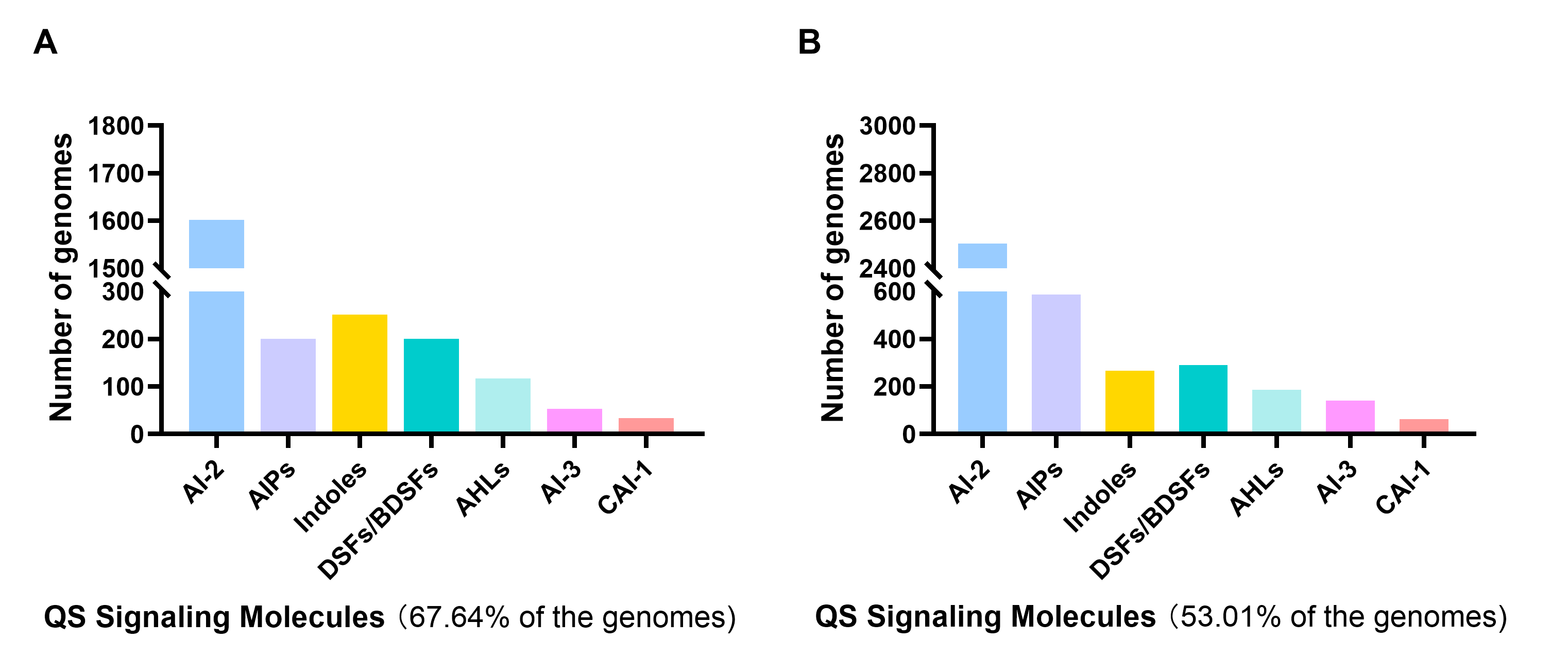

Supplement: Supplementary file 6 — Additional file 5: Figure S1. The presence of putative QS molecule-related proteins in microbial genomes from different sources. (A) Putative QS molecule-related proteins in the rumen ecosystem, including cattle, sheep, moose, deer, and bison (as the control group). (B) Putative QS molecule-related proteins in the pig gut (as the negative control group). [file 40168_2022_1367_MOESM5_ESM.tif]

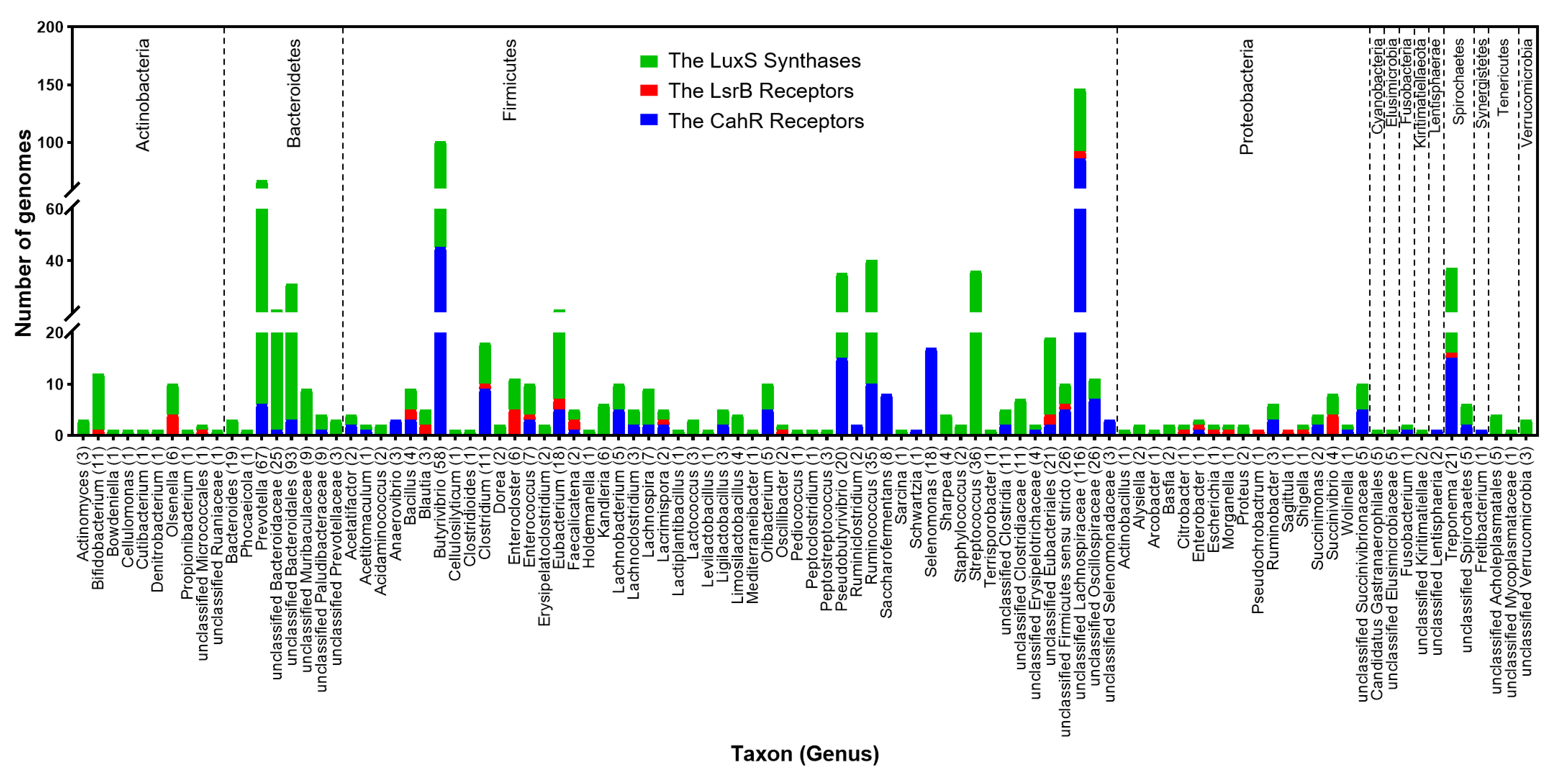

Supplement: Supplementary file 7 — Additional file 6: Figure S2. Distribution and abundance of AI-2 synthases and receptors based on QS in the rumen bacterial genomes. Numbers indicate the number of bacterial genomes in which the corresponding proteins were found at the genus level. The LuxS synthases, LsrB receptors, and CahR-type receptors were discovered within 88, 27, and 42 genera, respectively. [file 40168_2022_1367_MOESM6_ESM.tif]

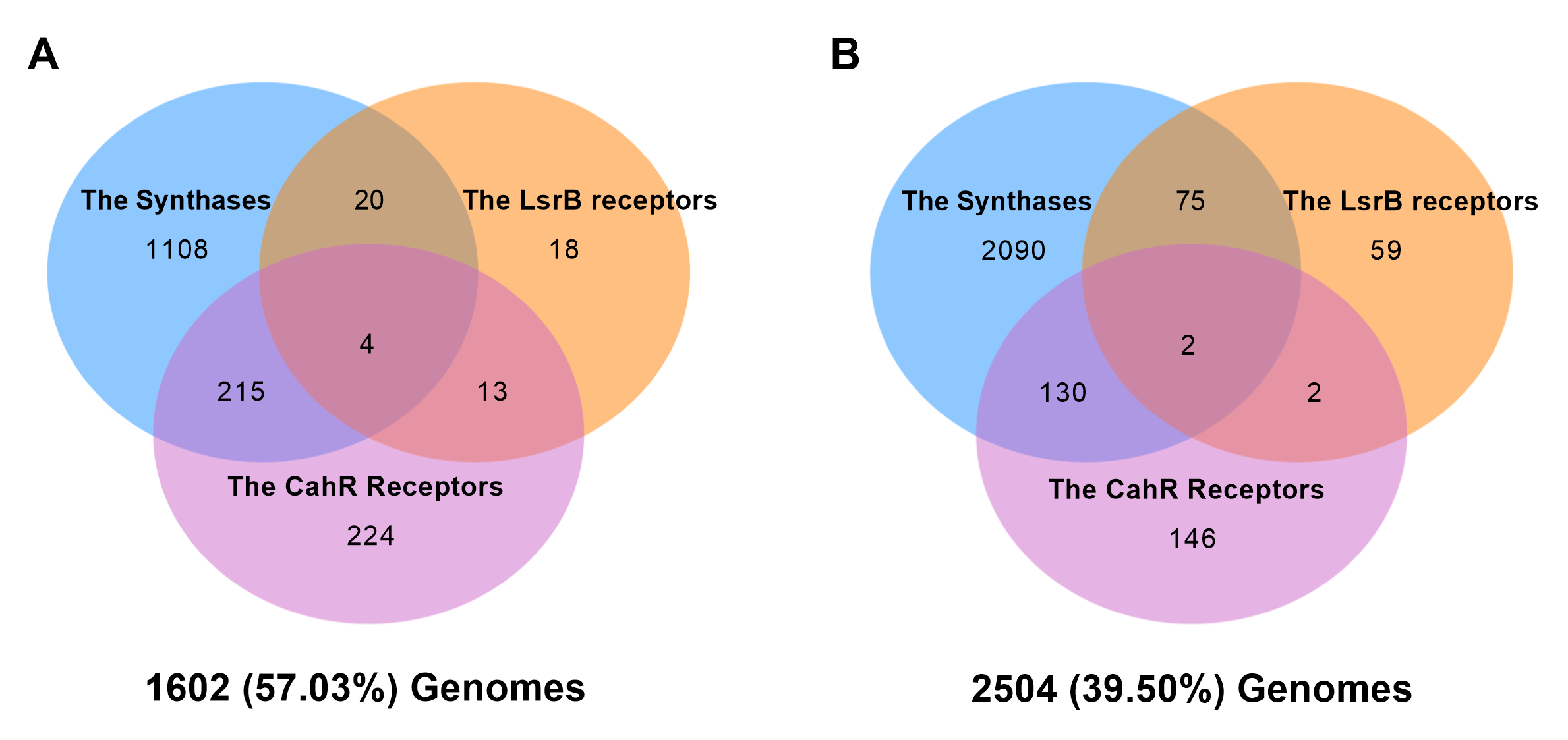

Supplement: Supplementary file 8 — Additional file 7: Figure S3. Occurrence of putative AI-2 synthase- and receptor-based QS in microbial genomes from different sources. (A) Putative LuxS synthases, LsrB, and CahR-type receptors in the rumen ecosystems including cattle, sheep, moose, deer, and bison (as the control group). (B) Putative LuxS synthases, LsrB, and CahR-type receptors in the pig gut (as the negative control group). Numbers indicate the numbers of bacterial genomes in which the corresponding proteins were found. [file 40168_2022_1367_MOESM7_ESM.tif]

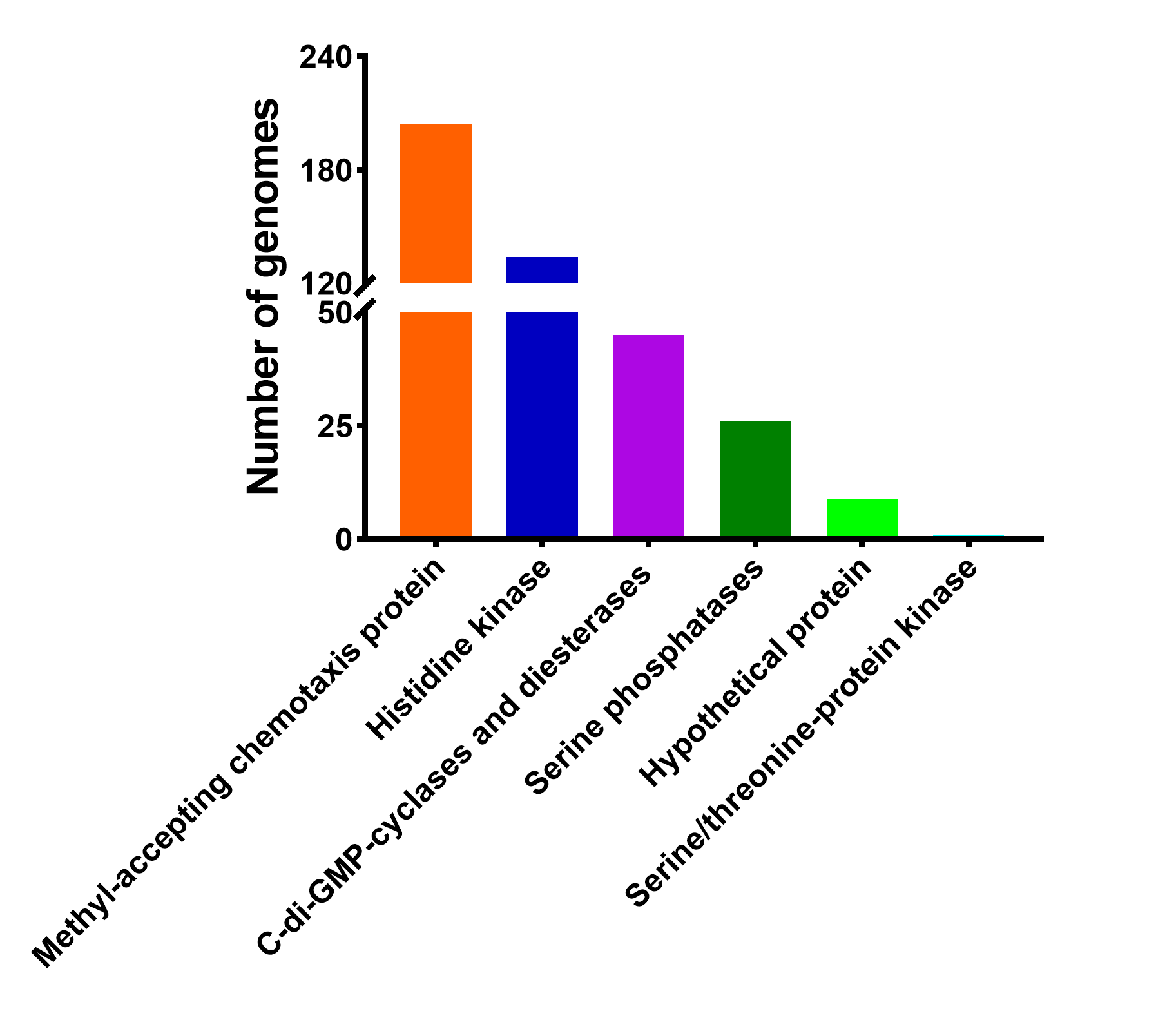

Supplement: Supplementary file 10 — Additional file 9: Figure S4. The types of the CahR-type receptors. Numbers indicate the number of bacterial genomes in which the corresponding proteins were found. The MCPs and HKs were two dominant AI-2 receptor proteins in rumen bacteria. [file 40168_2022_1367_MOESM9_ESM.tif]

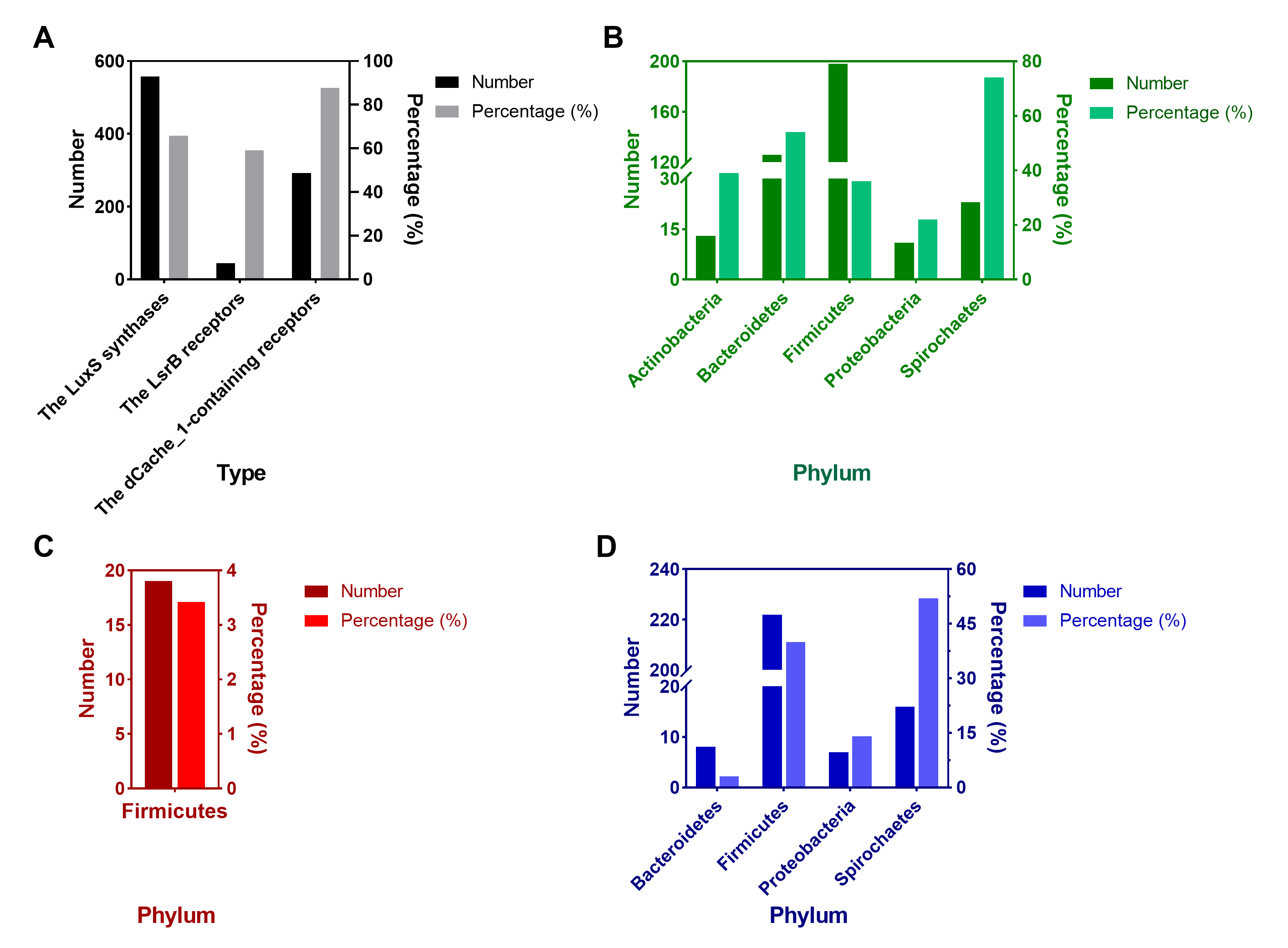

Supplement: Supplementary file 12 — Additional file 11: Figure S5. Expression of the predicted AI-2 synthases and receptors within a rumen microbial metatranscriptome at the phylum level. (A) The number of bacterial genomes containing AI-2-related genes and the percentage of corresponding genomes that expressed these genes. (B) The number of bacterial genomes containing luxS synthases and the percentage of the corresponding genomes that expressed luxS genes at the phylum level. (C) The number of bacterial genomes containing lsrB receptors and the percentage of corresponding genomes that expressed lsrB genes at the phylum level. (D) The number of bacterial genomes containing CahR-type receptor genes and the percentage of the corresponding genomes that expressed these genes at the phylum level. Numbers indicate the numbers of bacterial genomes in which the corresponding functions were found at the phylum level. The phyla (> 0.6% of total microbial genomes) were selected. A total of 380 (68% of 558,) genomes expressed luxS synthases, which were mainly distributed in the phyla Bacteroidetes and Firmicutes; 26 (59% of the 44) genomes expressed known receptor lsrB genes; and 256 genomes (88% of 292) expressed CahR-type receptors, which were largely located in the phyla Firmicutes and Spirochaetes. [file 40168_2022_1367_MOESM11_ESM.tif]

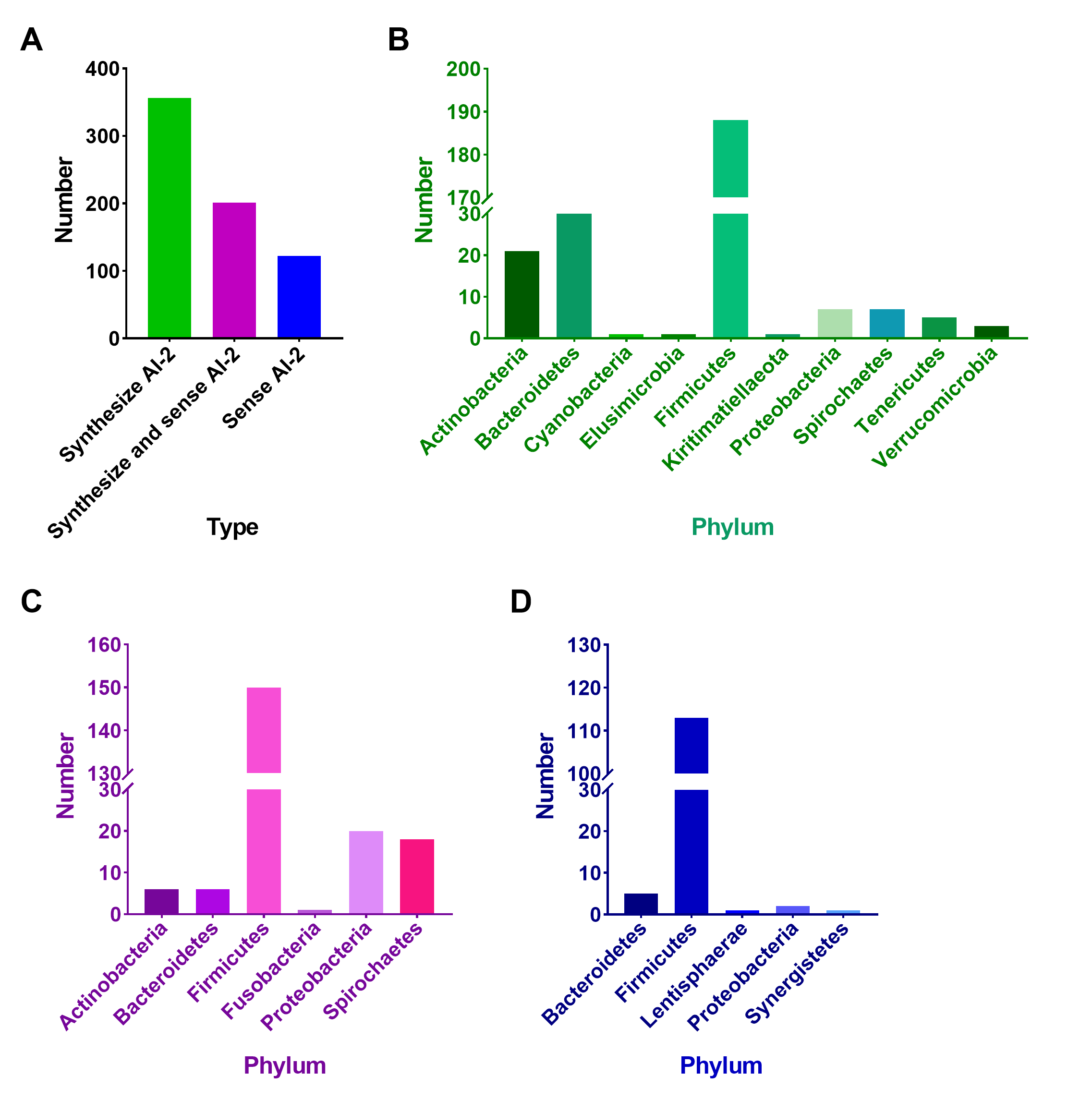

Supplement: Supplementary file 13 — Additional file 12: Figure S6. Widespread occurrence of rumen bacteria in the communication network. (A) The occurrence of rumen bacteria in the network. (B) The occurrence of bacteria that synthesize AI-2 at the phylum level. (C) The occurrence of bacteria that synthesize and sense AI-2 at the phylum level. (D) The occurrence of bacteria that sense AI-2 at the phylum level. Numbers indicate the numbers of bacterial genomes in which the corresponding functions were found at the phylum level. A total of 357 bacterial species were responsible for the synthesis of AI-2, which were mainly distributed in Actinobacteria, Bacteroidetes, and Firmicutes; 122 bacterial species sensed AI-2 to reprogram the expression of multiple genes, which were mainly distributed in Firmicutes; 201 bacterial species could not only produce AI-2 but also sense AI-2 in the environment, which primarily functioned in Firmicutes. [file 40168_2022_1367_MOESM12_ESM.tif]

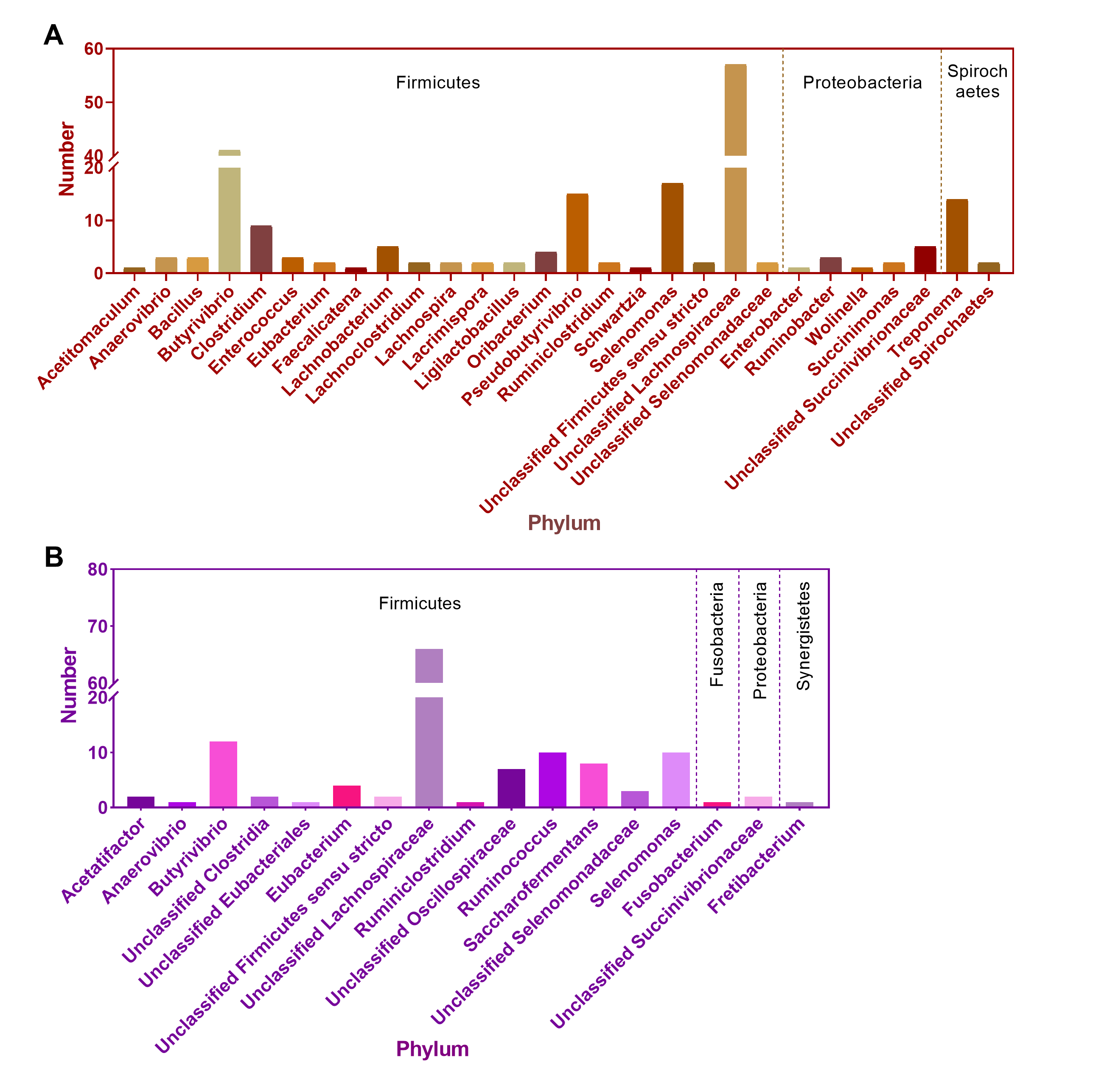

Supplement: Supplementary file 14 — Additional file 13: Figure S7. Widespread occurrence of MCPs and HKs as dominant AI-2 receptors in communication networks. [file 40168_2022_1367_MOESM13_ESM.tif]
